# Dorsal parietal area 5 only encodes the immediate reach in sequential arm movement

**DOI:** 10.1186/1471-2202-13-S1-P160

**Published:** 2012-07-16

**Authors:** Yuhui Li, He Cui

**Affiliations:** 1 Brain and Behavior Discovery Institute, Georgia Health Sciences University, Augusta, GA 30912, USA; 2 Department of Psychiatry and Health Behavior, Georgia Health Sciences University, Augusta, GA 30912, USA

## 

To generate an action sequence, it is essential to integrate information regarding all temporally coordinating motor elements. Spatial information about both immediate and subsequent component reaches is encoded simultaneously in the parietal reach region (PRR) [1]. However, it is still unclear how a cognitive sequence conveying multiple goals in parallel is discomposed into series of discrete movement commands to be executed by the musculoskeletal system.

In the present study, we recorded single-neuron activity from dorsal area 5 (area 5d) in the posterior parietal cortex while monkeys performed a memory-guided double-reach task. Briefly, the monkey was required to touch a fixation center at the trial beginning. Then, the first and second goals were simultaneously displayed for 400ms with a square and a triangle (shifted counter-clockwise from the square by 135°), respectively. After a 600ms delay, the center dot dimmed (GO signal) and the monkey was allowed to initiate the reaching sequence to touch the locations previously cued by the square and triangle in the correct order. Single-reach trials were pseudo-randomly interleaved with the double-reach trials for a control.

Among all 98 task-related cells we recorded from two monkeys, 35 (36%), 75 (77%) and 75 (77%) exhibited significant directional tuning during the delay (0~400ms before GO), pre-movement (0~200ms before the onset of the 1^st^ movement), and peri-movement (0~200ms after the onset of the 1^st^ movement) periods, respectively. In all above groups, the tuning curves in double-reach were similar to those in the single-reach trials (*p* > 0.05). To quantitatively analyze tuning dynamics, we calculated the vector sum of the neuronal activity in a 200ms sliding window across all directions as the instantaneous preferred direction. Prior to the offset of the 1^st^ movement, there was no significant difference in the preferred directions between single- and double-reach. Thereafter, the preferred direction in double-reach trials gradually rotated counter-clockwise toward the second reaching goal. Around 100ms before the onset of the 2^nd^ movement, the shift saturated at 135° at the population level, co-varying with the switch of immediate reaching movements.

We calculated the Fano Factor (FF, as the variance versus mean of the spike counts) across trials in each movement-direction/cell combination with a 100ms sliding window. In contrast to numerous other cortical sensorimotor regions [2], area 5d activity exhibited low variability (FF = ~1.0) and the FF changed modestly during the whole preparatory period. Although FF increased sharply after GO signal (up to ~1.3), this tendency could be weaken by aligning the spike trains to the movement onset (reduce to ~1.1) or by separating fast and slow reaction trials (reduce to ~1.1-1.2 only). This is generally the case regardless of aligning to the onset of the movement in the single reach trials, or 1^st^ or 2^nd^ movements in the double-reach trials.

The results that area 5d activity only conveys information about the immediate reach with low variance stick to motor execution suggest that area 5d may play a key role in translating cognitive action sequences into executive motor commands to activate muscle synergies.
